# A Lightweight CNN for Multiclass Retinal Disease Screening with Explainable AI

**DOI:** 10.3390/jimaging11080275

**Published:** 2025-08-15

**Authors:** Arjun Kumar Bose Arnob, Muhammad Hasibur Rashid Chayon, Fahmid Al Farid, Mohd Nizam Husen, Firoz Ahmed

**Affiliations:** 1 Department of Computer Science, American International University-Bangladesh, Dhaka 1229, Bangladesh; arjunkumarbosu@gmail.com (A.K.B.A.); chayon@aiub.edu (M.H.R.C.); 2 Faculty of Computer Science and Informatics, Berlin School of Business and Innovation, 12043 Berlin, Germany; 3 Malaysian Institute of Information Technology, Universiti Kuala Lumpur, Kuala Lumpur 50250, Malaysia

**Keywords:** convolutional neural network, diabetic retinopathy, eye disease, fundus imaging, retinal disease classification

## Abstract

Timely, balanced, and transparent detection of retinal diseases is essential to avert irreversible vision loss; however, current deep learning screeners are hampered by class imbalance, large models, and opaque reasoning. This paper presents a lightweight attention-augmented convolutional neural network (CNN) that addresses all three barriers. The network combines depthwise separable convolutions, squeeze-and-excitation, and global-context attention, and it incorporates gradient-based class activation mapping (Grad-CAM) and Grad-CAM++ to ensure that every decision is accompanied by pixel-level evidence. A 5335-image ten-class color-fundus dataset from Bangladeshi clinics, which was severely skewed (17–1509 images per class), was equalized using a synthetic minority oversampling technique (SMOTE) and task-specific augmentations. Images were resized to 150×150 px and split 70:15:15. The training used the adaptive moment estimation (Adam) optimizer (initial learning rate of $1\times 10^{-4}$, reduce-on-plateau, early stopping), $\ell_{2}$ regularization, and dual dropout. The 16.6 M parameter network converged in fewer than 50 epochs on a mid-range graphics processing unit (GPU) and reached 87.9% test accuracy, a macro-precision of 0.882, a macro-recall of 0.879, and a macro-F1-score of 0.880, reducing the error by 58% relative to the best ImageNet backbone (Inception-V3, 40.4% accuracy). Eight disorders recorded true-positive rates above 95%; macular scar and central serous chorioretinopathy attained F1-scores of 0.77 and 0.89, respectively. Saliency maps consistently highlighted optic disc margins, subretinal fluid, and other hallmarks. Targeted class re-balancing, lightweight attention, and integrated explainability, therefore, deliver accurate, transparent, and deployable retinal screening suitable for point-of-care ophthalmic triage on resource-limited hardware.

## 1. Introduction

The global situation of eye disease today is that it is a significant public health issue, and an estimated 2.2 billion people suffer from some kind of vision loss or eye disease [1]. Cataracts remain a leading cause of blindness, and access to care remains an issue, even with more eye-care programs [2]. Age-related macular degeneration (AMD) is also increasing, with an estimated increase in its prevalence from 196 million in 2020 to 288 million by 2040 [3]. Despite the decline in the prevalence of blindness and vision impairment, the number of people affected is growing as a result of population growth and aging [4]. Furthermore, the comorbidity of eye diseases with other disease states, such as diabetes and cardiovascular diseases, delineates the need for integrated care models [1]. These issues require action across both public health policies and eye-care provisions to facilitate universal access to prevention and treatment [4].

Socioeconomic determinants contribute to the global burden of eye disease, and several studies have reported disparities in prevalence and economic costs. For example, AMD is extremely expensive economically, with costs of €43.2 billion in the United States (US) alone, largely due to lost productivity and decreased well-being, disproportionately affecting older individuals and those in less privileged socioeconomic groups [5]. Similarly, the burden of glaucoma in DALYs increases in populations with lower human development indices (HDIs) and mean years of schooling (MYS), whose socioeconomic status is negatively related to disease burden [6]. Blindness and visual impairment due to limited access to adequate interventions also occur more frequently in countries with a low HDI, a result that corroborates the link between socioeconomic development and eye outcomes [7]. The increasing burden of near-vision impairment, particularly in low-income and middle-income countries, strongly underscores the need for targeted public health interventions to address these disparities [8]. Socioeconomic determinants are active contributors to global eye disease trends, and interdisciplinary measures are required to safeguard against them.

Convolutional neural networks (CNNs) may lead the way when it comes to the diagnosis of eye disease because they are capable of processing and analyzing large groups of retinal images in a manner that supports rapid and effective disease diagnosis, like that of diabetic retinopathy and glaucoma. Some notable reasons are the application of advanced image preprocessing techniques, such as data augmentation and generative adversarial networks (GANs), for the enhancement of the image quality and data imbalance correction, respectively, to enhance the performance of the model [9]. In addition, CNNs are supported by end-to-end data-driven approaches that facilitate the auto-learning of discriminative features of inexplicably high-dimensional medical images using conventional practices [10]. The use of diversified deep architecture models, such as hybrid architectures, improves diagnostic accuracy and efficacy, with a diagnostic accuracy of above 80% in some studies [11]. The problems of overfitting and heterogeneous datasets remain the foremost drivers of innovativeness in such diagnostic equipment [12].

While standard deep learning models have shown promise, their direct clinical application is often hindered by critical, practical barriers. First, large, computationally expensive models are impractical for deployment in point-of-care or low-resource settings, where the diagnostic need is often greatest. Second, the “black box” nature of many advanced models, where the reasoning behind a diagnosis is unclear, erodes trust, and makes it difficult for ophthalmologists to verify or accept automated results. Finally, real-world clinical datasets are inherently imbalanced, causing models to perform poorly on rarer but equally critical diseases, which can lead to missed diagnoses.

This study is, therefore, motivated by the urgent need for a solution that overcomes these specific application-focused challenges. We propose a lightweight CNN architecture designed not only for high accuracy but also for computational efficiency, enabling its use on resource-limited hardware. Crucially, by integrating explainable artificial intelligence (XAI) techniques such as gradient-based class activation mapping (Grad-CAM), we provide transparent visual evidence for each diagnosis, fostering the clinical trust and validation required for adoption. By addressing data imbalance with targeted oversampling using the synthetic minority oversampling technique (SMOTE) and standard data augmentation techniques, we ensured that the model is reliable across a wide spectrum of retinal conditions. The ultimate goal is to bridge the gap between artificial intelligence (AI) potential and practical clinical utility, delivering a screening tool that is accurate, efficient, and trustworthy for real-world ophthalmic triage. The main objectives are the following:

- Designing an optimal CNN-based classifier for 10 retinal diseases with data augmentation and class balancing.
- Applying real-time explainability using gradient-based attention mapping (Grad-CAM and Grad-CAM++) for clinical transparency.
- Developing a reproducible pipeline for clinical AI deployment with class balancing included and with SMOTE and computational efficiency considered.

The remainder of this paper is organized as follows: Section 2 contains related studies on eye disease diagnosis approaches using deep learning; Section 3 presents the overall research plan and proposed approach for eye disease classification; the results and in-depth analysis of the proposed model and baseline models are presented in Section 4; finally, Section 5 contains the overall verdict, limitations, and future work.

## 2. Related Studies

Several recent studies have focused on nonstandard modalities and methodologies for eye disease diagnosis, taking advantage of developments in machine learning and imaging technologies to improve patient care and accuracy. They refer to the ability of deep learning models to process retinal images for the diagnosis of diabetic retinopathy and age-related macular degeneration with highly accurate results.

According to a systematic review, deep learning systems have considerably boosted the classification and diagnosis of various eye diseases in contemporary studies. Deep learning systems, that is, CNNs, have been deployed in a vast range of imaging modalities, such as optical coherence tomography (OCT) and fundus photographs, to maximize the diagnostic efficiency and accuracy for diabetic retinopathy, glaucoma, and age-related macular degeneration [12,13]. For example, one experiment derived from the Ocular Disease Intelligent Recognition (ODIR) database recorded an 89.64% test accuracy using the MobileNet model, and one experiment exceeded 90% accuracy in OCT image binary classification [14,15]. Despite these advances, several challenges remain to be resolved, such as the variability of data and access to large-scale heterogeneous datasets required to enhance the robustness and interoperability of models mentioned by Dash et al. [12]. Future directions involve integrating multimodal imaging and patient metadata to further increase model performance and clinical usefulness [16].

Imbalanced eye disease images can significantly impact the performance of the classification model and require effective data-balancing techniques. SMOTE is a popular technique for generating synthetic examples of minority classes by interpolation with neighboring points, but it is prone to overgeneralization and noise sensitivity [17,18]. Mohammed et al. mentioned that variants such as FADA-SMOTE resolve these problems through minority instance clustering and synthetic sample generation optimization to reduce overlap with majority classes [19]. Safe-Level-SMOTE is yet another variant of the original SMOTE that adds a “safe level” that prefers sampling in areas with fewer majority instances to improve classification accuracy [20]. In addition, techniques such as SMOTE-LMVDE incorporate noise detection and local mean adaptive vectors into synthetic sample generation, thereby outperforming conventional methods [21]. All of these developments provide a strong foundation for the handling of class imbalance in eye disease imagery.

New developments in CNNs for the detection of retinal diseases have highlighted the strengths of diverse architectures for diagnosing diabetic retinopathy (DR) and glaucoma. Thakoor et al. points to the strength of their CNN models for glaucoma detection using optical coherence tomography images through transfer learning and ensemble techniques for performance improvement as well as explainability [22]. Abushawish et al. [23] provides a detailed overview of CNNs for DR diagnosis, tracing the evolution from conventional techniques to deep learning and the significance of explainability through mechanisms such as Grad-CAM. Pandey et al. [24] demonstrates how a stack of CNNs achieves a higher accuracy of identifying a number of retinal conditions from fundus images than board-certified ophthalmologists at an average rate of 79.2% to the latter’s 72.7%, whereas for human clinicians. Furthermore, the effectiveness of deep CNNs has been validated, and the most reliable architecture, EfficientNetB4, has achieved high training accuracy [25,26]. All these studies confirm the potential of CNNs in enhancing the diagnostic efficiency and accuracy of retinal disease detection.

Data augmentation methods play a crucial role in increasing the accuracy of eye disease image classification models by solving problems such as small datasets and overfitting. For instance, Moya-Sánchez et al. [27] demonstrated that their specific augmentation technique improved the classification accuracy by as much as 9% for non-mydriatic fundus images, demonstrating the necessity for specific augmentation techniques for specific images. Goceri et al. [28] emphasized that the performance of the augmentation techniques is dependent on the disease and imaging method and, thus, needs to be selected accordingly in order to provide excellent performance. Furthermore, generative modeling approaches, as investigated in glaucoma classification, have achieved outstanding improvements in sensitivity, specificity, and overall accuracy, thereby highlighting the contributions of various image qualities during training, as mentioned by Leonardo et al. [29]. In addition, Mounsaveng et al. [30] proposed a bi-level optimization method that can automatically search for augmentation parameters with performance rivaling or even surpassing conventional approaches. Taken together, the aforementioned studies show that successful data augmentation is essential for enhancing the robustness and performance of deep learning models for medical image classification [31].

The clinical adoption of AI in ophthalmology has numerous significant challenges and limitations. Despite AI demonstrating high accuracy in the diagnosis of diseases such as diabetic retinopathy and glaucoma, AI systems are often affected by data variability and the need for large and diverse datasets to ensure confidence in good performance across populations [32]. Additionally, the interpretability of AI models is an issue of concern because the majority of models are “black boxes,” and clinicians find it hard to interpret their decision making [12]. In addition, overdependence on automation would lead to the deskilling of medical professionals who would overdepend on AI systems for diagnosis [33]. Other limitations include the incorporation of AI into existing workflows, the requirement for high-quality imaging data, and the risk of misdiagnosis caused by natural variability in clinical presentations [34,35]. These limitations must be overcome before AI technology can be effectively applied in ophthalmology.

While the reviewed literature highlights significant progress, our analysis identifies three interlinked constraints that previous studies often address in isolation, impeding the broad clinical deployment of automated retinal screening: the excessive computational demands of conventional CNNs, opaque, “black-box” models that erode clinician trust, and performance biases from imbalanced datasets. The primary distinction of our study lies in addressing these three challenges simultaneously through a unified framework. Instead of adapting heavyweight ImageNet encoders, we propose a custom, computationally efficient network that combines depthwise separable convolutions with dual squeeze-and-excitation (SE) and global-context (GC) attention modules. This architecture is specifically designed to maintain high discriminative power while remaining suitable for resource-limited point-of-care hardware. Furthermore, our framework does not treat explainability as a separate, post hoc analysis, but as a core requirement. The inference loop is intrinsically coupled with Grad-CAM and Grad-CAM++ to provide pixel-level visual evidence for every prediction, directly addressing the critical barrier of clinical trust. This synergistic combination of a lightweight architecture, integrated explainability, and robust data balancing provides a holistic solution tailored for real-world clinical viability and constitutes the main contribution and distinction of this work.

## 3. Methodology

**Figure 1:**
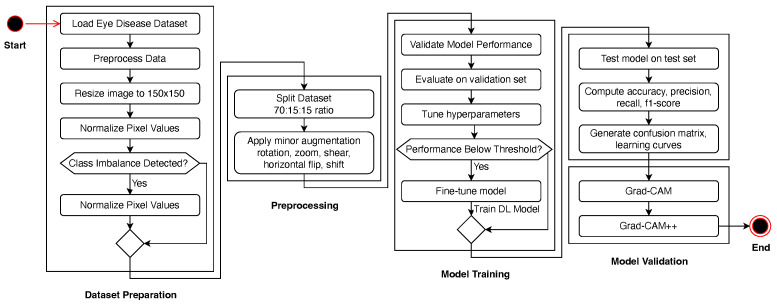
Proposed research methodology pipeline.

The research pipeline is illustrated in Figure 1, which shows the linear workflow from dataset preprocessing to model assessment. It starts with dataset preparation, where images are resized, normalized, and balanced using SMOTE if a class imbalance exists. The dataset was divided into training (70%), validation (15%), and testing (15%) datasets. To enhance the generalization of the model, we performed a set of data augmentation methods, including rotation, zoom, shear, and flipping. We then trained the CNN-based deep learning model with optimized hyperparameters, such as Adam optimization and learning-rate scheduling. The model was then validated, and fine-tuning was performed if the trained model failed to exceed a specified threshold. Upon obtaining good performance, the best model was assessed on the test dataset against accuracy, precision, recall, and F1-score measures.

### 3.1. Dataset Overview

**Figure 2:**
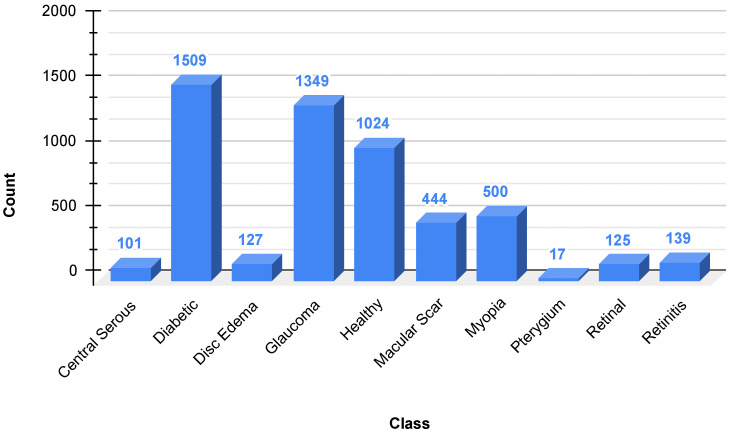
Dataset overview.

The data utilized in this study [36] are a dataset of 5335 color fundus images collected from two hospitals in Bangladesh with ten different classes of eye diseases, including Diabetic Retinopathy, Glaucoma, Macular Scars, and normal eyes, among others. As is evident in Figure 2, the dataset is extremely imbalanced, with Diabetic Retinopathy (1509 images) and Glaucoma (1349 images) being the most prominent, and classes such as Pterygium (17 images) and Central Serous Chorioretinopathy (101 images) being rare. This imbalance can bias model performance towards majority classes, and techniques such as SMOTE are needed to negate disparities. The photographs were captured using Topcon fundus cameras, resized to a uniform resolution of 2004 × 1690 pixels, and manually annotated by healthcare professionals for accuracy. The heterogeneity and clinical significance of the dataset render it a practical resource for the training of sturdy deep learning algorithms for the automated detection of eye diseases, provided that geographical and ethnic limitations are considered for generalizability.

### 3.2. Preprocessing

The data for this study included images divided into different eye disease conditions. There are several operations in the preprocessing pipeline, including image loading, normalization, class balancing, dataset splitting, and data augmentation.

The images were loaded from their directory and resized to a uniform size of 150×150 pixels to maintain consistency in all samples. All images were transformed into an array format, and labels were assigned accordingly based on their respective classes. The pixel values were normalized between [0, 1] by dividing the pixel value by 255 for smooth model training.

**Figure 3:**
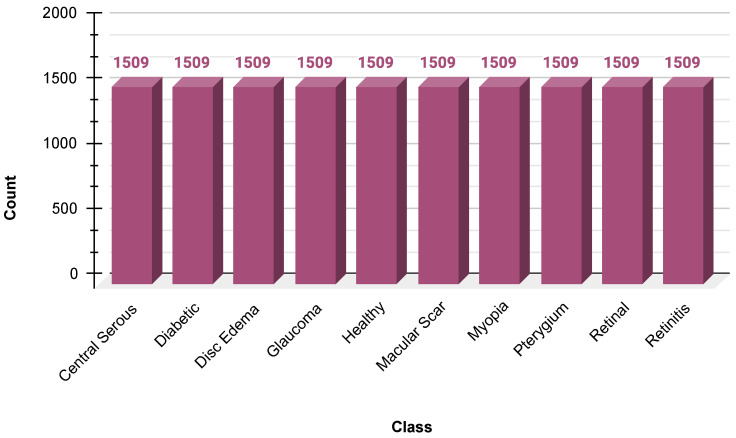
Dataset overview after applying SMOTE.

Because there was an inherent skew in the dataset, SMOTE was used to create synthetic samples of minority classes. SMOTE was used in the feature space after flattening the image arrays to ensure a balanced distribution of the class before reshaping the images to their original dimensions. This helped minimize the bias toward majority classes, along with model generalization. The data distribution after SMOTE is shown in Figure 3.

**Figure 4:**
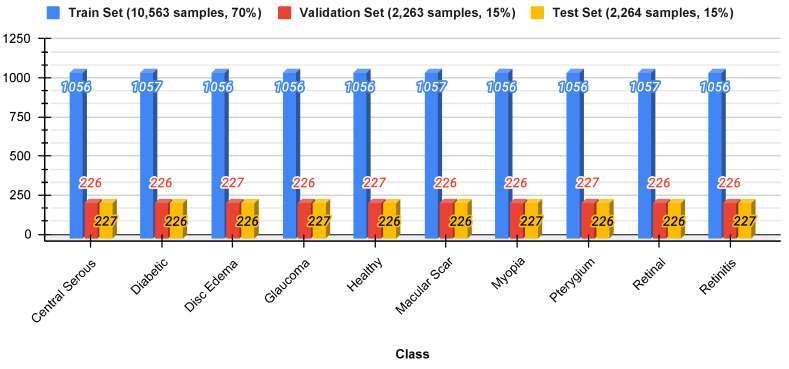
Dataset distribution.

To ensure appropriate testing, the data were divided into three sets: 70% for training, 15% for validation, and 15% for testing. Stratified sampling was used to preserve the original class distribution in each subset to prevent data leakage and ensure a fair representation of all classes. Figure 4 shows the distribution of the split datasets.

**Figure 5:**
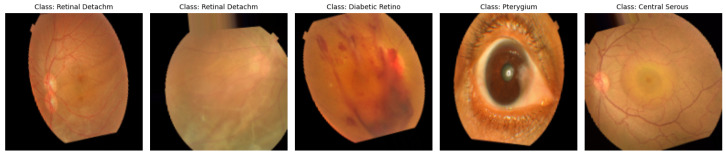
Random augmented sample images.

Data augmentation techniques were employed to enhance the generalization abilities of the model. Random transformations, such as rotation (up to 20°), width and height shift (up to 10%), shear transformation, zooming, and horizontal flip, were performed to create variability in the training data. This augmentation procedure virtually adds diversity to the dataset, prevents overfitting, and makes the model more robust. The augmented images are shown in Figure 5.

### 3.3. Proposed Model

**Figure 6:**
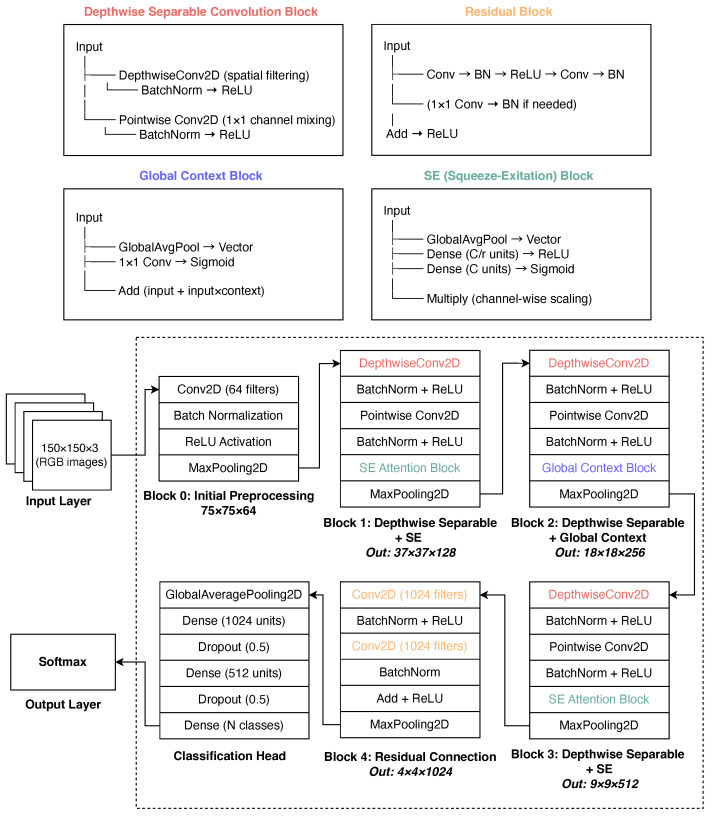
Proposed model architecture for multiclass eye-disease image classification.

The deep learning method proposed in this study, presented in Figure 6, is a high-level hierarchical feature extraction network for classifying medical images. The network started with an input layer accepting 150×150×3 RGB images, and the first convolutional block consisted of a 3×3 convolution with 64 filters, batch normalization, ReLU activation, and 2×2 max pooling. Four subsequent feature extraction blocks integrate complementary attention mechanisms: blocks 1 and 3 combine depthwise separable convolutions with squeeze-and-excitation (SE) attention for channel-wise recalibration; block 2 couples depthwise separable convolutions with global-context (GC) attention to capture long-range dependencies; block 4 employs a residual connection to facilitate gradient flow. Max-pooling progressively reduces spatial dimensions while expanding the channel width (64→128→256→512→1024).

The network terminates in a global average pooling (GAP) classification head with two dense layers regularized by $\ell_{2}$ weight decay and dropout (p=0.5). A softmax layer produces class probability distributions. The training employs Adam (initial learning rate of $10^{-4}$) combined with reduce-on-plateau scheduling and early stopping. This design balances the computational efficiency (via depthwise separable convolutions) with expressive attention mechanisms, allowing the model to focus selectively on diagnostically relevant regions across multiple feature hierarchies [37].

- **Notation:** Let the input image be $x\in R^{150\times 150\times 3}$ and let $X\in R^{H\times W\times C}$ denote an intermediate feature map [38], where *H* and *W* are the spatial dimensions and *C* is the channel dimension. The operator BN(·) is batch normalization [39], ϕ(·)=max(0,·) is ReLU activation [40], and $\sigma \left(\cdot \right)=1\;/\;\left(1+e^{-(\cdot )}\right)$ is the logistic sigmoid. Global average pooling (GAP) [41] is defined as
  (1) $$\mathrm{GAP}\left(X\right)=\frac{1}{HW}\sum_{i=1}^{H}\sum_{j=1}^{W}X_{i,j,:}\in R^{C},$$
  and ⊙ denotes the channel-wise (Hadamard) product.
- **Depthwise separable convolution:** For a 3×3 kernel footprint $K={\left\{-1,0,1\right\}}^{2}$, input $X\in R^{H\times W\times C}$ and depthwise kernel $K^{\mathrm{dw}}\in R^{3\times 3\times C}$ [42,43],
  (2) $$\begin{array}{cc} Y_{i,j,c}^{\mathrm{dw}} & =\sum_{(p,q)\in K}K_{p,q,c}^{\mathrm{dw}}\;X_{i+p,j+q,c}, \end{array}$$
  (3) $$\begin{array}{cc} {\mathrm{DSConv}}_{C\;\to F}\left(X\right) & =\phi \;\left(\mathrm{BN}\;\left({\mathrm{Conv}}_{1\times 1}^{C\to F}\left(\phi (\mathrm{BN}\left(Y^{\mathrm{dw}}\right))\right)\right)\right), \end{array}$$
  where *F* is the number of output channels of the pointwise (1×1) convolution.
- **Attention modules (r=16):**
  (4) $$\begin{array}{cc} \mathrm{SE}(X) & =\sigma \;\left(W_{2}\;\phi \left(W_{1}\;\mathrm{GAP}\left(X\right)\right)\right)⊙X, \end{array}$$
  (5) $$\begin{array}{cc} \mathrm{GC}(X) & =\left(1+\sigma (W_{g}\;\mathrm{GAP}\left(X\right))\right)⊙X, \end{array}$$
  where $W_{1}\in R^{\frac{C}{r}\times C}$, $W_{2}\in R^{C\times \frac{C}{r}}$, and $W_{g}\in R^{C\times C}$ are trainable weight matrices [44].
- **Residual block (stride s=1, output channels *F*):**
(6) $$\begin{array}{cc} U & =\phi \;\left(\mathrm{BN}({\mathrm{Conv}}_{3\times 3}^{F,s}\left(X\right))\right), \end{array}$$
(7) $$\begin{array}{cc} U & =\mathrm{BN}\left({\mathrm{Conv}}_{3\times 3}^{F,1}\left(U\right)\right), \end{array}$$
(8) $$\begin{array}{cc} S & =\mathrm{BN}\left({\mathrm{Conv}}_{1\times 1}^{F,s}\left(X\right)\right), \end{array}$$
(9) $$\begin{array}{cc} {\mathrm{ResBlock}}_{F}\left(X\right) & =U+S. \end{array}$$
- **End-to-end computation:** Setting $X^{\left(0\right)}=x$ and letting MP denote 2×2 max-pooling (stride 2),
  (10) $$\begin{array}{cc} X^{\left(1\right)} & =\mathrm{MP}\;\left(\phi \left(\mathrm{BN}({\mathrm{Conv}}_{3\times 3}^{64}\left(X^{\left(0\right)}\right))\right)\right), \end{array}$$
  (11) $$\begin{array}{cc} X^{\left(2\right)} & =\mathrm{MP}\left(\mathrm{SE}\left({\mathrm{DSConv}}_{64\;\to 128}\left(X^{\left(1\right)}\right)\right)\right), \end{array}$$
  (12) $$\begin{array}{cc} X^{\left(3\right)} & =\mathrm{MP}\left(\mathrm{GC}\left({\mathrm{DSConv}}_{128\;\to 256}\left(X^{\left(2\right)}\right)\right)\right), \end{array}$$
  (13) $$\begin{array}{cc} X^{\left(4\right)} & =\mathrm{MP}\left(\mathrm{SE}\left({\mathrm{DSConv}}_{256\;\to 512}\left(X^{\left(3\right)}\right)\right)\right), \end{array}$$
  (14) $$\begin{array}{cc} X^{\left(5\right)} & =\mathrm{MP}\left({\mathrm{ResBlock}}_{1024}\left(X^{\left(4\right)}\right)\right). \end{array}$$
  Global average pooling yields $v=\mathrm{GAP}\left(X^{\left(5\right)}\right)\in R^{1024}$, followed by
  (15) $$\begin{array}{cc} h_{1} & ={\mathrm{Drop}}_{0.5}\;\left(\phi (W_{1}^{\mathrm{fc}}v+b_{1}^{\mathrm{fc}})\right), \end{array}$$
  (16) $$\begin{array}{cc} h_{2} & ={\mathrm{Drop}}_{0.5}\;\left(\phi (W_{2}^{\mathrm{fc}}h_{1}+b_{2}^{\mathrm{fc}})\right), \end{array}$$
  (17) $$\begin{array}{cc} p\; & =\mathrm{softmax}\left(W_{o}h_{2}+b_{o}\right)\in R^{N}, \end{array}$$
  where $W_{1}^{\mathrm{fc}}\in R^{1024\times 512}$, $W_{2}^{\mathrm{fc}}\in R^{512\times 512}$, $W_{o}\in R^{N\times 512}$, and *b* are the bias vectors.
- **Loss function (batch size *B*, classes *N*):**
  (18) $$L\left(\theta \right)=-\frac{1}{B}\sum_{n=1}^{B}\log p_{y_{n}}\left(x_{n};\theta \right)+\lambda {∥\theta ∥}_{2}^{2},\;\lambda =0.01,$$
  where θ collects all trainable parameters. Optimization uses Adam with an initial learning rate of $10^{-4}$, a reduce-on-plateau schedule (factor 0.5, patience 5), and early stopping (patience of 15, restoring the best weights).

The complete architectural and training configurations of the proposed model are presented in Table 1. Table 2 summarizes the parameter counts of the proposed model. With roughly 16.6 million parameters (approximately 63 MB), of which more than 99.9% are trainable, the model is compact enough for a single mid-range GPU while still providing sufficient representational capacity for the target classification task. To provide a clear, quantitative context for our lightweight design, Table 3 compares the approximate total parameter counts of our proposed architecture against the standard baseline models used for benchmarking in Section 4. This comparison highlights the structural efficiency of our model, which is a core component of its design for point-of-care applications.

### 3.4. Evaluation Metrics

To assess the performance of the proposed deep learning model for multiclass classification of eye diseases, several evaluation metrics were employed [45].

- **Accuracy:** This represents the overall correctness of the model across all classes. It is computed as
  (19) $$\mathrm{Accuracy}=\frac{TP+TN}{TP+TN+FP+FN}$$
  where TP, TN, FP, and FN denote true positives, true negatives, false positives, and false negatives, respectively.
- **Precision:** This measures the proportion of correctly predicted positive observations to the total predicted positive observations:
  (20) $$\mathrm{Precision}=\frac{TP}{TP+FP}$$
- **Recall (Sensitivity):** This indicates the ability of the model to correctly find all the relevant cases (true positives) for each class:
  (21) $$\mathrm{Recall}=\frac{TP}{TP+FN}$$
- **F1-score:** This is a harmonic mean of the precision and recall, used to balance the two, especially in cases of class imbalance:
  (22) $$F1-\mathrm{score}=2\cdot \frac{\mathrm{Precision}\cdot \mathrm{Recall}}{\mathrm{Precision}+\mathrm{Recall}}$$
- **Confusion Matrix:** This is a tabular representation that outlines the performance of a classification model by comparing actual versus predicted classes, allowing for detailed per-class error analysis.
- **Training Time:** The total computational time taken to train the model until early stopping or completion of all epochs. This provides insights into the efficiency and scalability of the model.

These metrics were calculated using classification_report and confusion_matrix functions from the scikit-learn library. Furthermore, the training and validation accuracy and loss curves over epochs were plotted to visualize the learning behavior of the model and identify possible overfitting. To allow fair benchmarking, the same metrics were used on a set of popular transfer-learning models (VGG16, ResNet50, InceptionV3, MobileNetV2, and EfficientNetB0). The results are summarized in a comparison table.

### 3.5. Explainable AI

While the lightweight model architecture detailed in Figure 6 addresses the critical challenge of computational efficiency and hardware deployability, it does not inherently address the equally important challenge of clinical trust. An accurate prediction is of limited value if the end-user, the ophthalmologist, cannot understand or verify the basis of the model’s decision. Therefore, the second key component of our proposed system is the integration of XAI to make the reasoning transparent. The lightweight and explainable components are designed to be complementary; the former makes the model deployable, and the latter makes it trustworthy. They are combined in the final workflow, where every prediction generated by the efficient model is accompanied by a visual saliency map, ensuring that the system is both practical for real-world use and interpretable for clinical validation.

Grad-CAM and Grad-CAM++ play an important role in the classification of eye diseases through the improvement of deep learning model interpretability in medical imaging, particularly fundus images. Grad-CAM provides heat maps that highlight regions of interest, such as glaucoma and retinal disease lesions, thereby enabling the easy localization and diagnosis of diseases [46,47]. For example, in the diagnosis of glaucoma, Grad-CAM has been applied in CNNs with high accuracy and ROC-AUC scores, thereby proving to be effective in image salient feature localization [48]. Grad-CAM++ is another enhancement of the original technique that generates more precise lesion localization, which is extremely useful when lesions are tiny and scattered, e.g., in retinal disease diagnosis [49]. This not only improves classification performance but also enables improved clinical decision making through visual explanations of model predictions [46,50].

## 4. Results and Analysis

**Figure 7:**
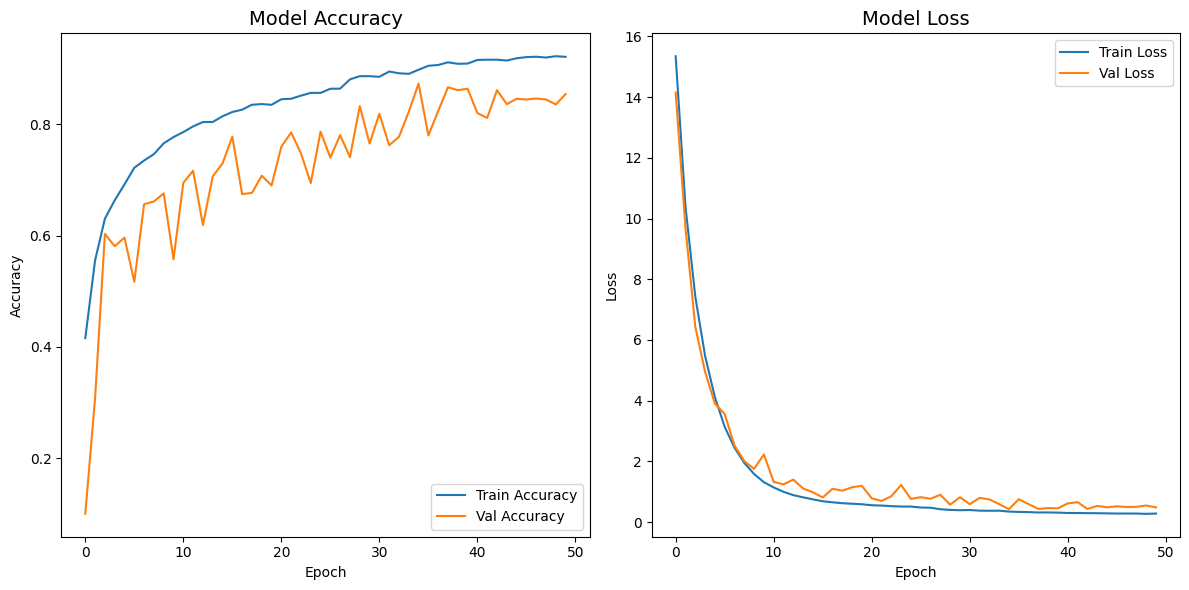
Training and validation accuracy and loss curves for the proposed model.

Figure 7 depicts the evolution of the accuracy and loss for both the training and validation splits over 50 epochs. The network exhibited a steep performance gain during the first ten epochs, with the training accuracy increasing from 34.7% to 78.6% and a five-fold drop in loss. The improvement then became more gradual, and a peak validation accuracy of 87.4% was achieved at epoch 35, where the corresponding validation loss decreased to 0.426. After that point, the validation metrics stabilized, whereas the training loss continued to decrease slightly, indicating that the reduce-on-plateau schedule (learning rate halved every five stagnant epochs) and dropout contained overfitting. Early stopping was triggered at epoch 50; however, the model parameters from epoch 35, saved by the model checkpoint callback, were ultimately restored, yielding a final test accuracy of 87.9% and a test loss of 0.417.

**Figure 8:**
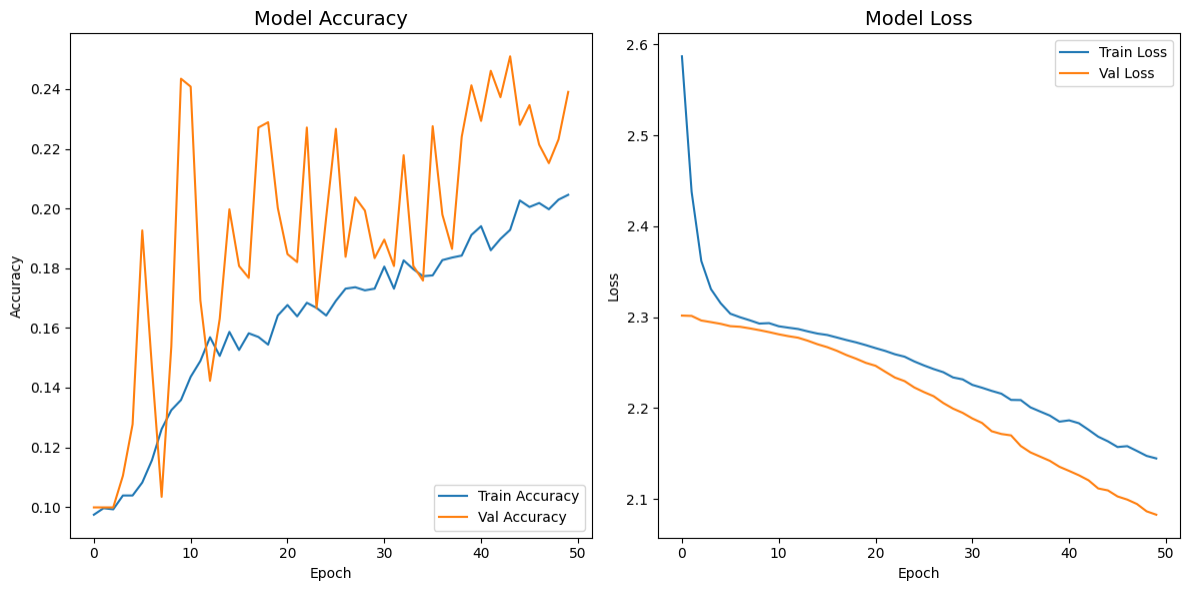
Learning curve of VGG16.

**Figure 9:**
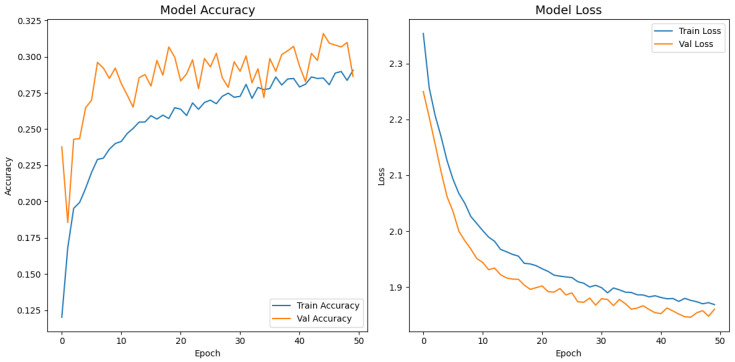
Learning curve of ResNet50.

**Figure 10:**
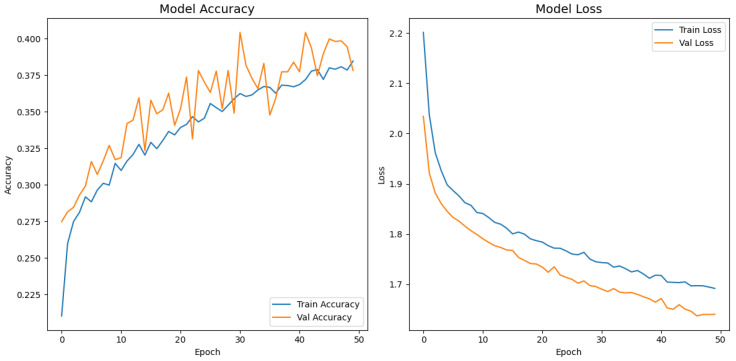
Learning curve of InceptionV3.

**Figure 11:**
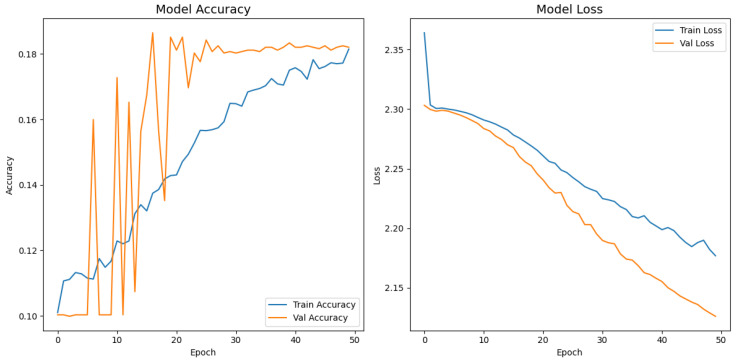
Learning curve of MobileNetV2.

**Figure 12:**
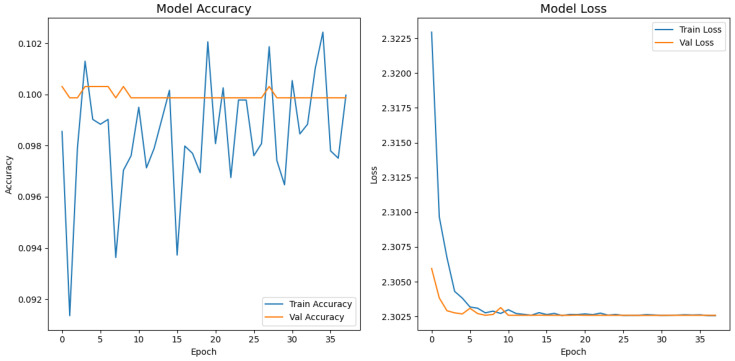
Learning curve of EfficientNetB0.

Figure 8, Figure 9, Figure 10, Figure 11 and Figure 12 display the training–validation accuracy (left) and loss (right) trajectories for the five ImageNet backbones after fine-tuning on the same ophthalmic split. All converged within approximately 15–20 epochs; however, their endpoint performances diverged sharply. MobileNetV2 and EfficientNetB0 plateau early with large train–val gaps, revealing under-fitting and heavy over-regularization, respectively. VGG16 and ResNet50 train longer but flatten far below the ceiling attained by InceptionV3. Even InceptionV3’s best validation accuracy (40.4%) was less than half of the 87.4% achieved by the proposed model, whose task-specific attention modules add discriminative power. It is critical to contextualize the performance of the baseline models. Their lower-than-expected accuracy is largely attributable to two deliberate experimental constraints. First, as noted, the models were constrained to a 150×150 input size, which differed from their original pretraining dimensions (e.g., 224×224). Second, the pretrained layers of these backbones were frozen and used as fixed feature extractors, with only the final classification layers being trained. Although fine-tuning the backbones would likely yield higher scores, our approach was chosen to ensure a fair comparison of all architectures under identical, computationally efficient conditions that simulate a resource-constrained deployment. Therefore, the results demonstrate that our custom architecture is more effective at extracting discriminative features under these specific lightweight constraints than the adapted standard backbones.

Table 4 confirms the following visual trends: InceptionV3 is the strongest baseline; however, it still trails the proposed model by more than 47 percentage points. MobileNetV2 and EfficientNetB0 had accuracies below 20% and 10%, respectively, indicating that extreme parameter compression degrades fine-grained recognition. Despite similar training times, the proposed model delivered the best accuracy–time ratio, achieving a relative error reduction of 58.1% over the top baseline. While the training times reported are comparable, this reflects the early stopping protocol, which concluded training for each model once its performance on the validation set plateaued. The primary advantage of a lightweight architecture in this context is not the reduced training time but rather its efficiency during inference. A model with fewer parameters, such as our proposed network, requires significantly less computational power and memory to perform a prediction. This efficiency enables deployment on resource-limited, point-of-care hardware, which is a key target of this study. The larger baseline models, despite similar training times on powerful research hardware, would have a much higher inference latency, making them less practical for real-time clinical triage.

**Figure 13:**
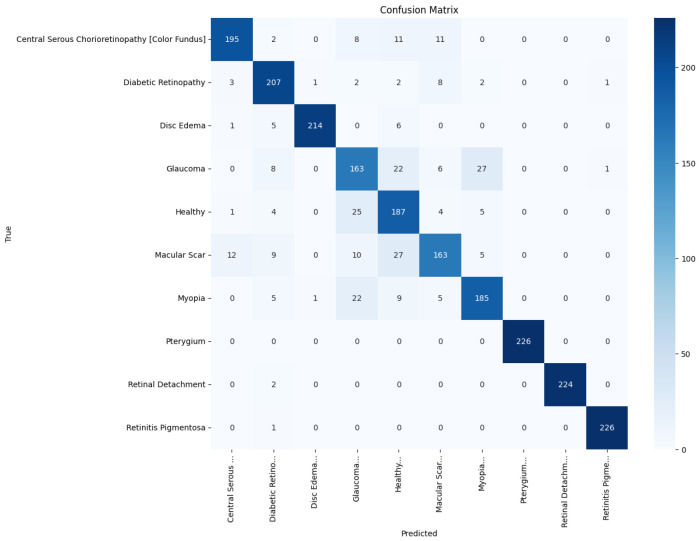
Confusion matrix of the proposed model.

**Figure 14:**
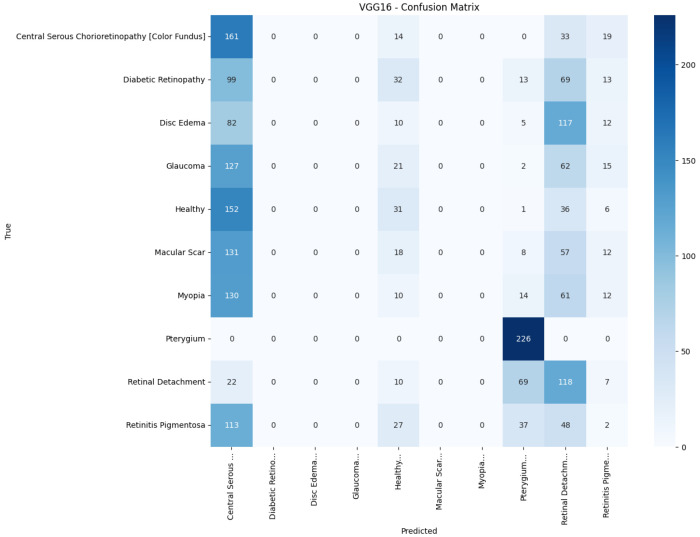
Confusion matrix of VGG16.

**Figure 15:**
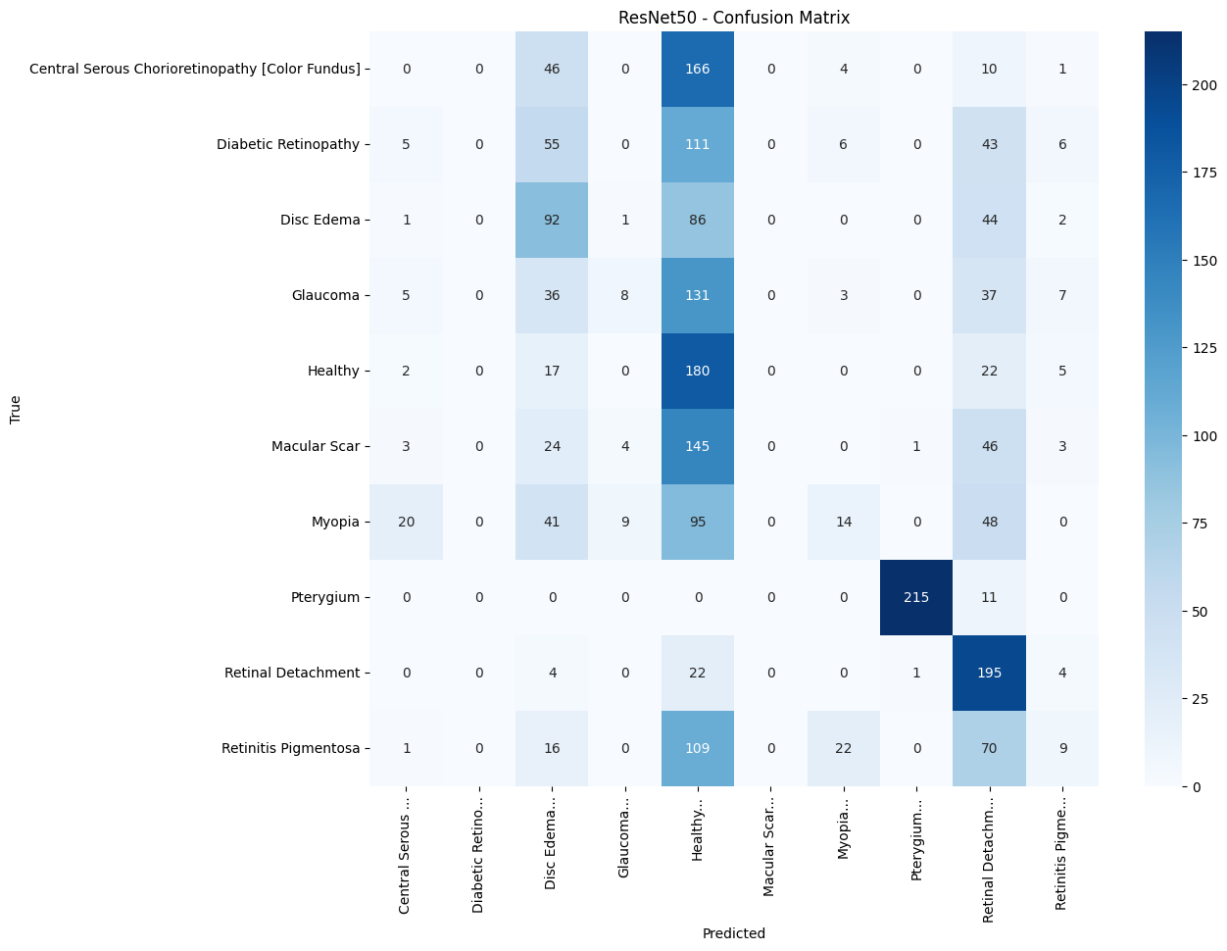
Confusion matrix of ResNet50.

**Figure 16:**
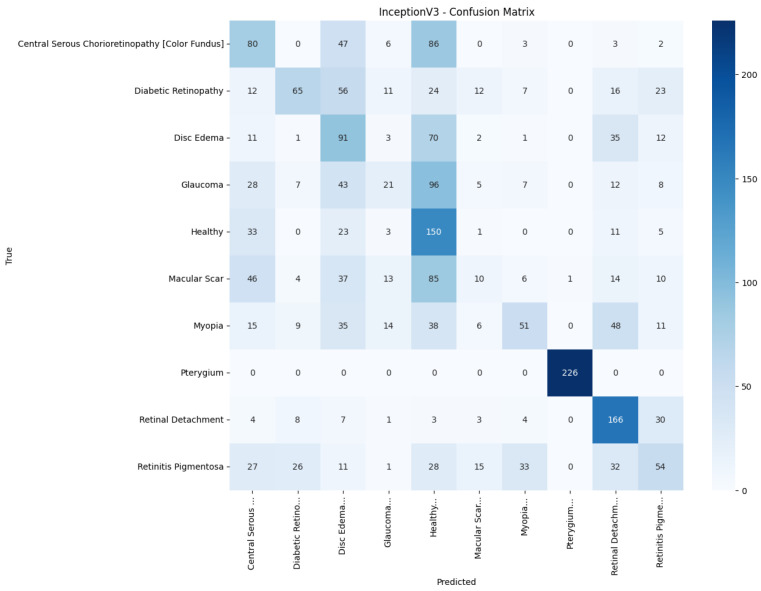
Confusion matrix of InceptionV3.

**Figure 17:**
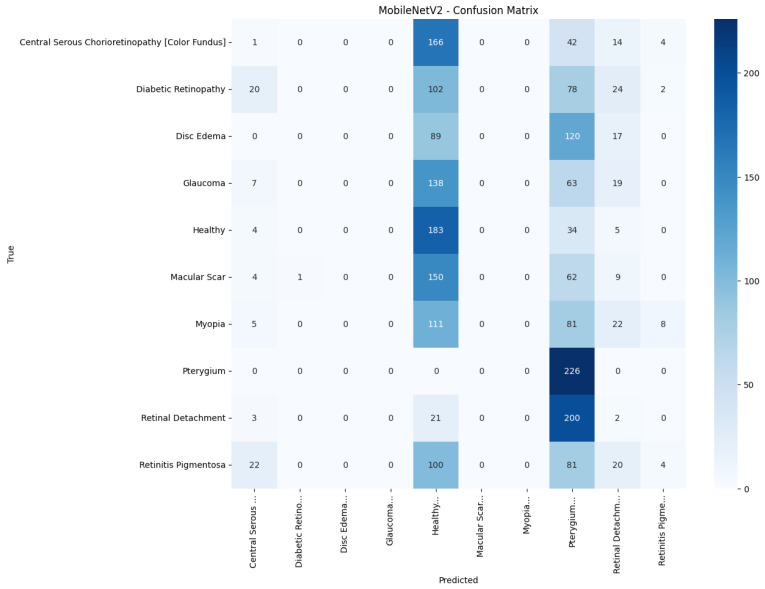
Confusion matrix of MobileNetV2.

**Figure 18:**
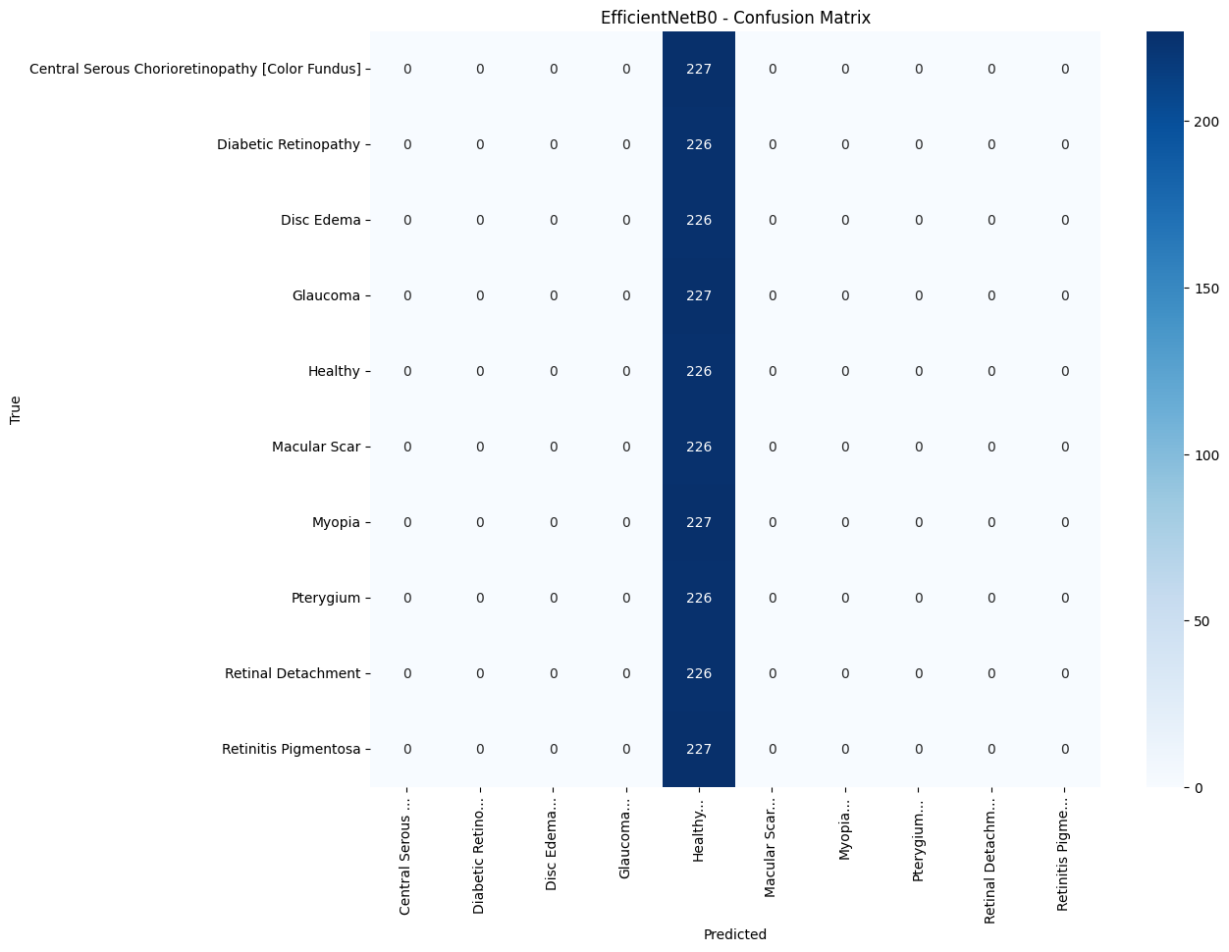
Confusion matrix of EfficientNetB0.

Figure 13, Figure 14, Figure 15, Figure 16, Figure 17 and Figure 18 visualize the classwise behaviour of every model by means of 10×10 confusion matrices. Several common trends have emerged. First, Pterygium (a conjunctival lesion with a distinctive wing-shaped profile) is almost never confused with any retinal disorder; every network, even EfficientNetB0, assigns the 226 test images of this class to the correct column. The opposite extreme is Central Serous Chorioretinopathy (Color Fundus); VGG16, ResNet50, and MobileNetV2 mislabel the majority of Color Fundus cases as Healthy or Glaucoma, reflecting subtle macular fluid that mimics physiological foveal reflexes. Across all baselines, the anatomically related triad of Color Fundus, Macular Scars, and Myopia form the densest off-diagonal blocks, indicating systematic confusion among macula-centric pathologies.

Model-specific observations reinforce the quantitative metrics in Table 4. EfficientNetB0 in Figure 18 collapses into a near-single-column predictor, assigning almost every image to the dominant class (healthy), which explains its accuracy of 10%. MobileNetV2 in Figure 17 retains some class discrimination but still confuses more than one-third of Disc Edema, Myopia, and Macular Scar images with unrelated categories. ResNet50 in Figure 15 reduces gross errors yet continues to misclassify about one quarter of Glaucoma as Healthy. InceptionV3 in Figure 16 exhibits the clearest diagonal among the baselines, but substantial leakage remains from the Color Fundus and Macular Scar into neighboring labels.

The confusion matrix of the proposed model in Figure 13 shows a markedly stronger diagonal and thinner off-diagonal than the baseline. True-positive counts exceeded 185 for eight of the ten classes, whereas the largest remaining confusion, 27 glaucoma images predicted as myopia, was less than one-sixth of the corresponding error in ResNet50. These patterns corroborate the aggregate improvements reported earlier and highlight that attention-guided depthwise design not only raises overall accuracy but also balances performance across clinically heterogeneous categories.

Table 5 presents the precision, recall, and F1-score for each pathology and model. The proposed model attains the highest value in every class, with F1-scores of at least 0.97 for Pterygium, Retinal Detachment, and Retinitis Pigmentosa, and balanced scores for the more challenging macular disorders: 0.89 for central serous chorioretinopathy (color fundus) and 0.77 for Macular Scars. The best baseline, InceptionV3, registers an F1-score below 0.60 in six of the ten classes and a macro-average of only 0.38. Lightweight architectures such as MobileNetV2 and EfficientNetB0 perform little better than chance, concentrating most predictions on the majority Healthy category and producing macro-F1-scores of 0.07 and 0.02, respectively. These results show that the proposed model improves class discrimination uniformly across the diagnostic spectrum rather than boosting accuracy by favoring only the largest or easiest classes.

**Figure 19:**
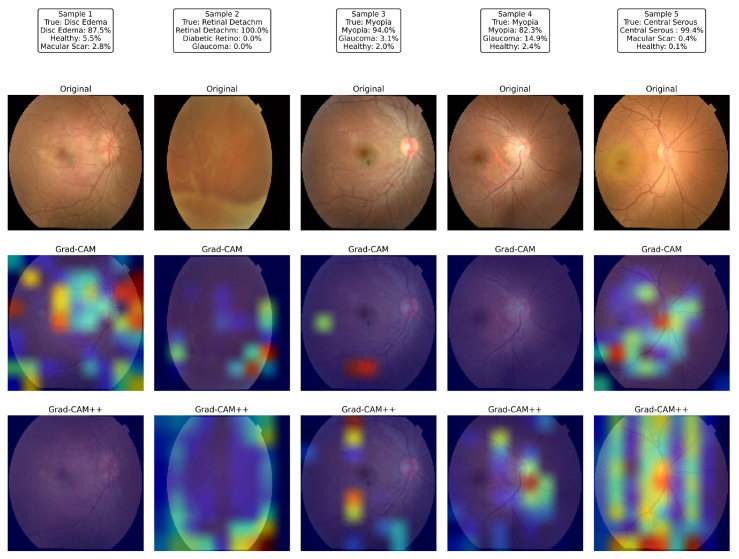
Visual explanations produced by the proposed model: For five representative fundus images (columns), the first row shows the original photograph, the second row shows the Grad-CAM heat-map, and the third row shows the corresponding Grad-CAM++ map, highlighting the disease-specific regions that drive the network’s predictions.

Figure 19 illustrates the visual explanations produced by the proposed network for five representative test images. For each sample, the first row shows the raw color fundus photograph, the second row superimposes a Grad-CAM saliency map, and the third row displays the corresponding Grad-CAM++ map, both computed from the last convolutional layer of the proposed model.

Sample 1 (disc edema) revealed strong activation around the swollen optic nerve head and peripapillary nerve fiber layer, exactly where neuro-ophthalmologists inspect for raised intracranial pressure. In Sample 2 (total retinal detachment), the network was concentrated on the superior nasal periphery, where the detached neuro-retina was most clearly elevated, ignoring the unaffected posterior pole; the detached fold coincided with the hottest Grad-CAM++ pixels. Samples 3 and 4, both labeled myopia, exhibit heatmaps centered on the tesselated fundus and tilted optic disc typical of high axial myopia while sparing the relatively featureless mid-periphery. In Sample 5, the model focused on the juxta-foveal serous blister characteristic of central serous chorioretinopathy; the elongated hotspot across the macula in Grad-CAM++ matched the subretinal fluid pocket observed by clinicians.

Across all cases, the Grad-CAM++ maps were sharper and more localized than the vanilla Grad-CAM overlays; however, both highlighted the same disease-specific structures, confirming that the attention mechanisms guided the classifier toward clinically meaningful regions rather than spurious background patterns. These interpretable heat maps strengthen the confidence in the proposed model’s predictions and underline its potential for real-world decision support.

## 5. Conclusions

This study addressed three persistent barriers that restrict the clinical adoption of automated retinal disease screening: pronounced class imbalance in publicly available datasets, computational demands of conventional convolutional backbones, and lack of transparent decision pathways in deep learning models. These issues collectively impede reliable performance, real-time deployment in low-resource settings, and clinician trust.

To overcome these obstacles, we designed a 16.6 M parameter CNN that integrates depthwise separable convolutions with squeeze-and-excitation and global-context attention modules. The training pipeline couples SMOTE with extensive geometric and photometric augmentation and optimizes the network using Adam with learning-rate scheduling and early stopping. Prediction transparency was provided by Grad-CAM and Grad-CAM++, ensuring that each classification was accompanied by a pixel-level saliency explanation.

Fulfilling the study’s primary objectives, the resulting model converged in fewer than 50 epochs on a single mid-range graphics processing unit and achieved 87.9% accuracy, a macro-precision of 0.882, a macro-recall of 0.879, and a macro-F1-score of 0.880 on a rigorously held-out ten-class color fundus test set. Relative to the strongest ImageNet baseline (Inception-V3, 40.4% accuracy), this represents a 58% reduction in error while sustaining a throughput suitable for point-of-care triage. True-positive rates exceeded 95% for eight disorders, and saliency maps consistently highlighted diagnostic retinal structures, thereby strengthening clinical interpretability and confidence.

Notwithstanding these advantages, this study has several limitations that warrant discussion. First, the evaluation was conducted on a retrospective dataset from a single national cohort; although rigorous, it lacks external validation using diverse international datasets. Therefore, the model’s robustness across different patient ethnicities, device manufacturers, and clinical settings is yet to be confirmed. Second, our model is currently limited to a single imaging modality (color fundus photographs) and performs disease classification without assessing the severity (e.g., grading diabetic retinopathy), which is a crucial step for clinical management. Finally, the diagnostic process relies solely on the image, without incorporating other rich clinical data such as patient history or intraocular pressure, which are integral to an ophthalmologist’s final assessment.

These limitations directly inform our directions for future work. A crucial next step is to perform external validation of the model on multi-ethnic, multi-device repositories and to conduct prospective clinical trials to evaluate its real-world performance and utility. We also plan to extend the model’s capabilities to include the severity grading of key diseases. To create a more powerful diagnostic tool, we will explore multimodal fusion techniques that integrate our image-based classifier with structured patient data. Furthermore, we will continue to incorporate other imaging modalities, such as volumetric OCT and ultrawide-field imaging. Finally, exploring advanced training paradigms, such as federated or self-supervised learning, could enhance data diversity and generalizability while preserving patient privacy. Addressing these aspects will advance the readiness of lightweight, explainable screening tools for equitable global eye-care delivery.

## Figures and Tables

**Table 1:** Model hyperparameters and training configuration.

Component	Specification
Architecture parameters	
Input dimensions	150×150×3
Initial convolution	64 filters, 3×3 kernel, ReLU
Block progression	[128, 256, 512, 1024] filters
Specialised layers	
Depthwise separable conv	Depthwise 3×3 + pointwise 1×1
SE block	Reduction ratio r=16
Global context block	Channel-wise attention ($W_{g}$)
Residual block	Two 3×3 convs, skip connection (s=1)
Classification head	
Dense layers	1024 → 512 units, ReLU
Dropout rates	0.5 (both layers)
$\ell_{2}$ regularization	λ=0.01
Output layer	Soft-max, *N* classes
Training configuration	
Optimizer	Adam ($\eta =10^{-4}$, β1=0.9, β2=0.999)
Batch size	32 (*B*)
Epochs	50 (early stopping)
Learning-rate schedule	Reduce on plateau (factor 0.5, patience 5)
Minimum learning rate	$10^{-6}$
Early stopping	Patience 15, restore best weights

**Table 2:** Parameter breakdown of the proposed model.

Parameter Class	Count	Memory Footprint
Trainable	16,552,114	63.14 MB
Non-trainable	8960	35.00 KB
Total	16,561,074	63.18 MB

**Table 3:** A comparison of model architecture parameter counts, where baseline parameters are for the convolutional base (include_top = False).

Model Architecture	Total Parameters (Ãpproximate)
VGG16	134.3 M
ResNet50	23.6 M
InceptionV3	21.8 M
Proposed Model	16.6 M
EfficientNetB0	4.1 M
MobileNetV2	2.3 M

**Table 4:** Performance comparison of baseline backbones versus the proposed model.

Model	Accuracy	Precision	Recall	F1-Score	Train Time (s)
EfficientNetB0	0.100	0.010	0.100	0.018	1826
MobileNetV2	0.184	0.065	0.184	0.071	2329
VGG16	0.238	0.116	0.238	0.146	2419
ResNet50	0.315	0.270	0.315	0.233	2404
InceptionV3	0.404	0.414	0.404	0.378	2391
**Proposed Model**	**0.879**	**0.882**	**0.879**	**0.880**	**2414**

Best value per row in bold.

**Table 5:** Per-class precision/recall/F1-score for all six models.

Class (Support)	Proposed Model	InceptionV3	ResNet50	VGG16	MobileNetV2	EfficientNetB0
Color Fundus (227)	**0.92/0.86/0.89**	0.31/0.35/0.33	0.00/0.00/0.00	0.16/0.71/0.26	0.02/0.00/0.01	0.00/0.00/0.00
Diabetic Retinopathy (226)	**0.85/0.92/0.88**	0.54/0.29/0.38	0.00/0.00/0.00	0.00/0.00/0.00	0.00/0.00/0.00	0.00/0.00/0.00
Disc Edema (226)	**0.99/0.95/0.97**	0.26/0.40/0.32	0.28/0.41/0.33	0.00/0.00/0.00	0.00/0.00/0.00	0.00/0.00/0.00
Glaucoma (227)	**0.71/0.72/0.71**	0.29/0.09/0.14	0.36/0.04/0.06	0.00/0.00/0.00	0.00/0.00/0.00	0.00/0.00/0.00
Healthy (226)	**0.71/0.83/0.76**	0.26/0.66/0.37	0.17/0.80/0.28	0.18/0.14/0.16	0.17/0.81/0.28	0.10/1.00/0.18
Macular Scar (226)	**0.83/0.72/0.77**	0.19/0.04/0.07	0.00/0.00/0.00	0.00/0.00/0.00	0.00/0.00/0.00	0.00/0.00/0.00
Myopia (227)	**0.83/0.81/0.82**	0.46/0.22/0.30	0.29/0.06/0.10	0.00/0.00/0.00	0.00/0.00/0.00	0.00/0.00/0.00
Pterygium (226)	**1.00/1.00/1.00**	1.00/1.00/1.00	0.99/0.95/0.97	0.60/1.00/0.75	0.23/1.00/0.37	0.00/0.00/0.00
Retinal Detachment (226)	**1.00/0.99/1.00**	0.49/0.73/0.59	0.37/0.86/0.52	0.20/0.52/0.29	0.02/0.01/0.01	0.00/0.00/0.00
Retinitis Pigmentosa (227)	**0.99/1.00/0.99**	0.35/0.24/0.28	0.24/0.04/0.07	0.02/0.01/0.01	0.22/0.02/0.03	0.00/0.00/0.00
**Macro-avg.**	**0.88/0.88/0.88**	0.41/0.40/0.38	0.27/0.32/0.23	0.12/0.24/0.15	0.07/0.18/0.07	0.01/0.10/0.02

Best value per row in bold. Support is the number of test images in each class.

## Data Availability

The dataset used in this research was created by Sharmin, S., Rashid, M. R., Khatun, T., Hasan, M. Z., and Uddin, M. S. It is publicly available in the Mendely Data Repository and can be accessed at: https://data.mendeley.com/datasets/s9bfhswzjb/1 (accessed on 12 April 2025).
